# Challenges for better care based on the course of maternal body mass index, weight gain and multiple outcome in twin pregnancies: a population-based retrospective cohort study in Hessen/Germany within 15 years

**DOI:** 10.1007/s00404-020-05440-6

**Published:** 2020-01-29

**Authors:** Julia Schubert, Nina Timmesfeld, Kathrin Noever, Birgit Arabin

**Affiliations:** 1 Clara Angela Foundation, Alfred-Herrhausen-Straße 44, 58455 Witten, Germany; 2 Clara Angela Foundation, Koenigsallee 36, 14193 Berlin, Germany; 3 Department of Obstetrics Charite Humboldt University Berlin, Berlin, Germany; 4 Center for Mother and Child, Philipps University Marburg, Baldingerstraße, 35033 Marburg, Germany; 5 Department for Medical Computer Science, Biometry and Epidemiology, Ruhr-University Bochum, 44780 Bochum, Germany

**Keywords:** Obesity, Gestational weight gain, Twin pregnancy, Preterm delivery, Caesarean delivery

## Abstract

**Introduction:**

Studies on maternal weight, gestational weight gain and associated outcomes in twin pregnancies are scarce. Therefore, we analyzed these items in a large cohort.

**Methods:**

Data from 10,603/13,725 total twin pregnancies from the perinatal database in Hessen, Germany between 2000 and 2015 were used after exclusion of incomplete or non-plausible data sets. The course of maternal and perinatal outcomes was evaluated by linear and logistic regression models.

**Results:**

The rate of twin pregnancies increased from 1.5 to 1.9% (*p* < 0.00001). Mean maternal age and pre-pregnancy weight rose from 31.4 to 32.9 years and from 68.2 to 71.2 kg, respectively (*p* < 0.001). The rates of women with a body mass index ≥ 30 kg/m^2^ increased from 11.9 to 16.9% with a mean of 24.4–25.4 kg/m^2^ (*p* < 0.001). The overall increase of maternal weight/week was 568 g, the 25th quartile was 419, the 75th quartile 692 g/week. The total and secondary caesareans increased from 68.6 to 73.3% and from 20.6 to 39.8%, respectively (*p* < 0.001). Rates of birthweight < 1500 g and of preterm birth < 28 and from 28 to 33 + 6 weeks all increased (*p* < 0.01). No significant changes were observed in the rates of stillbirth, perinatal mortality and NICU admissions.

**Conclusion:**

The global trend of the obesity epidemic is equally observed in German twin pregnancies. The increase of mean maternal weight and the calculated quartiles specific for twin pregnancies help to identify inadequate weight gain in twin gestations. Policy makers should be aware of future health risks specified for singleton and twin gestations.

## Introduction

The globally observed obesity epidemic also affects women at childbearing age [1] with either singleton or twin pregnancies. Maternal overweight and obesity have been shown to increase adverse maternal and neonatal *short-term outcomes* such as gestational diabetes (GDM) and hypertension in pregnancy (HDP), preeclampsia, protracted labor, caesarean delivery as well as congenital malformations, stillbirth, large and small for gestational age fetuses (LGA/SGA), shoulder dystocia and neonatal hypoglycaemia [2, 3]. Even more worrisome are the consequences for *long-term health* such as persistent maternal obesity, earlier rates of maternal metabolic and cardiovascular diseases and for the children—up to adulthood—earlier rates of metabolic syndrome and even earlier death rates [4]. However, most of the data derive from singleton pregnancies. For twin pregnancies, rates and risk factors of maternal body mass index (BMI) and gestational weight gain (GWG) are scarce.

A systematic review from 2014 concluded that GWG in twin pregnancies is “a neglected area of research” [5]. When the Institute of Medicine (IOM, now “National Academy of Medicine”) established guidelines for weight gain during pregnancies in 2009, the recommendations for twin pregnancies were only provisional and did not include references for underweight women or for weekly weight gain [6]. Only a few publications reported on excessive gestational weight gain (EGWG) in twin gestation defined by provisional definitions [7, 8]. EGWG was then associated with higher rates of HDP and preeclampsia, but lower rates for low birthweight (BW).

In 2003, the twinning rates varied between 9.86/1000 in Japan [9] up to 30.1/1000 in the United States (US) [10, 11], but all countries still report on increasing twinning rates [9–12]. Therefore, the purpose of this study was to describe the twinning rates within one representative federal state (Hessen) in Germany. More importantly, we have strived to illustrate changes of maternal BMI, GWG in absolute grams/week and quartiles as well as available associated outcomes during 15 years within this large German Perinatal cohort between 2000 and 2015.

## Materials and methods

This study was based on data from the perinatal registry of hospital deliveries in Hessen, Germany, a federal state with 6,176,172 inhabitants in 2015 [13]. Access for this research project was kindly provided by the office for quality management in Hessen, Germany, for the years 2000 up to 2015. The raw data were anonymously offered with respect to patients and centers, but extensive plausibility controls had still to be performed to exclude cases where relevant data were missing or not plausible. Data from twin pregnancies were only included when maternal weight was given, maternal height was ≥ 120 cm and an early ultrasound examination had confirmed the gestational age, all before 14 gestational weeks. Deliveries < 24 gestational weeks, unknown maternal age or twins with unknown sex were excluded.

Examined maternal outcomes were: age, GWG in g/week and grouped as quartiles, BMI, HDP, caesarean delivery (total, secondary, emergency and only in the second twin) and postpartum haemorrhage (PPH, defined as a blood loss > 1000 ml).

The BMI was calculated as kg/m^2^ and classified according to the WHO criteria: BMI < 18.5 kg/m^2^: “underweight”; BMI 18.5–24.9 kg/m^2^: “normal weight”; BMI 25.0–29.9 kg/m^2^: “overweight”; BMI ≥ 30 kg/m^2^: “obesity” [14]. The difference between the maternal weight at the first examination and before delivery was divided by the duration of the interval in weeks to calculate the mean maternal GWG/week and associated quartiles, whereby the quartiles above 25% and below 75% were defined as “normal”.

The incidence of HDP was indirectly calculated from clinical findings according to the definition by the International Society for the Study of Hypertension in Pregnancy (ISSHP) [15].

Gestational age at birth, BW, stillbirth, perinatal mortality (death at birth or within 7 days), neonatal intensive care unit (NICU) admissions, an APGAR score value below 7 after 5 min, a pH of the umbilical artery below 7.1 and a combination of the APGAR score and the pH of the umbilical artery were examined as neonatal outcomes.

The BW was categorized into five groups: below 1500 grams (g), 1500–1999 g, 2000–2499 g, 2500–2999 g and ≥ 3000 g. Similarly, the gestational age at birth was categorized into three groups of preterm birth: < 28, 28 + 0 to 33 + 6 and 34 + 0 to 36 + 6 gestational weeks.

Significant differences during the observation period were analyzed using a linear regression model for numeric outcomes or a logistic regression model for categorical outcomes. The programs for statistical evaluation were R for Windows 3.5.1, R Studio (Version 1.1.456) and Excel 2013. In all figures with relative results, the absolute numbers for each specific items were added.

## Results

**Fig. 1:**
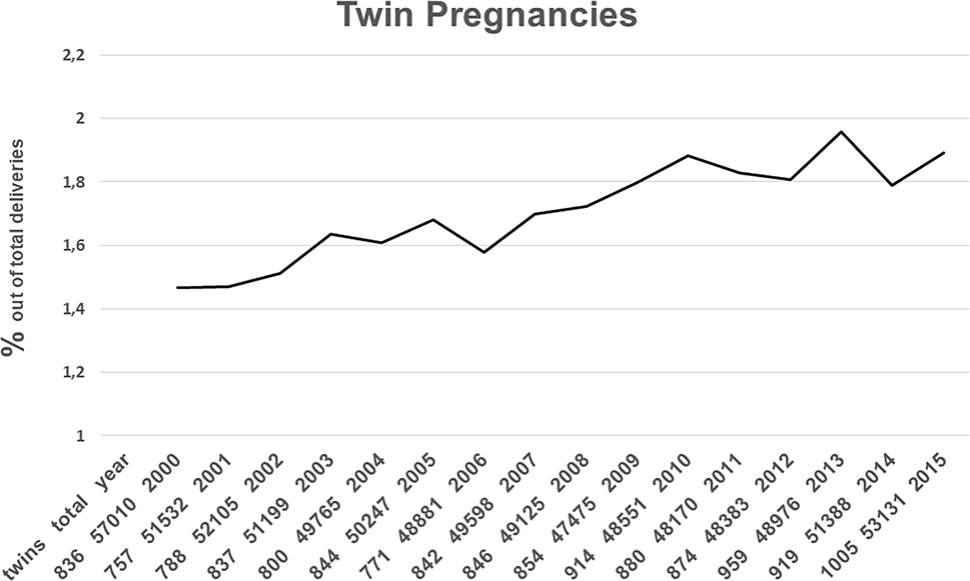
Course of twin pregnancy rates in Hessen 2000–2015, twins = number of twin pairs, total = total deliveries, *n* = 13,725/805,536 deliveries, *p* < 0.00001+, statistical analyses according to logistic regression

Between 2000 and 2015, 13,725 twin pregnancies of a total of 805,536 deliveries including all singleton, twin and multiple pregnancies were registered. This resulted in a mean twinning rate of 1.7% rising from 1.5% in 2000 to 1.9% in 2015, as shown in Fig. 1 (*p* < 0.00001). After the application of inclusion and exclusion criteria data sets from 10,603 twin pregnancies (77.3%) remained for further analyses.

The characteristics of the study group are shown in Table 1. More than 55% of the women were primiparous, about 38% did not work when registering and roughly 10% did not have a partner. Among the twin pairs, 36% had different sexes, and same sex twins showed an even distribution between males and females.

**Table 1:** Characteristics of the study group (*n* = 10,603/13,725 total twin deliveries between 2000 and 2015 in Hessen, Germany), 3122 pregnancies excluded after plausibility control

Characteristics	Mean (SD) or *n* (%)
Maternal age (years)	32.1 (5.09)
Maternal weight at 1st examination (kg)	69.6 (15.2)
Maternal height (cm)	167 (6.39)
Maternal BMI at 1st examination (kg/m^2^)	24.8 (5.10)
Week of 1st examination	8.24 (1.90)
Gestational age at birth (weeks)	35.8 (2.80)
Sex of twins	
Male/female	3831 (36.1%)
Female/female	3382 (31.9%)
Male/male	3390 (32.0%)
Mother without partner^a^	
Yes	871 (9.93%)
No	7896 (90.1%)
Mother’s origin	
German	8621 (81.3%)
Other nationalities	1982 (18.7%)
Previous deliveries^b^	
0	5995 (56.8%)
1	3213 (30.4%)
2	922 (8.74%)
3 or more	422 (4.00%)
Work^c^	
Housewife (at start of pregnancy)	2846 (38.1%)
Still in education	151 (2.02%)
Worker	225 (3.01%)
Employee	2711 (36.3%)
Academic position	1538 (20.6%)

*SD* standard deviation, *n* absolute number

^a^Missing values 1836

^b^Missing values 51

^c^Missing values 3132

The mean maternal weight at the first examination increased from 68.2 kg in 2000 to 71.2 kg in 2015 (*p* < 0.001), whereas the mean maternal height did not change (*p* = 0.58). Consequently, the early mean maternal BMI increased from 24.4 kg/m^2^ in 2000 to 25.4 kg/m^2^ in 2015 (*p* < 0.0001).

**Fig. 2:**
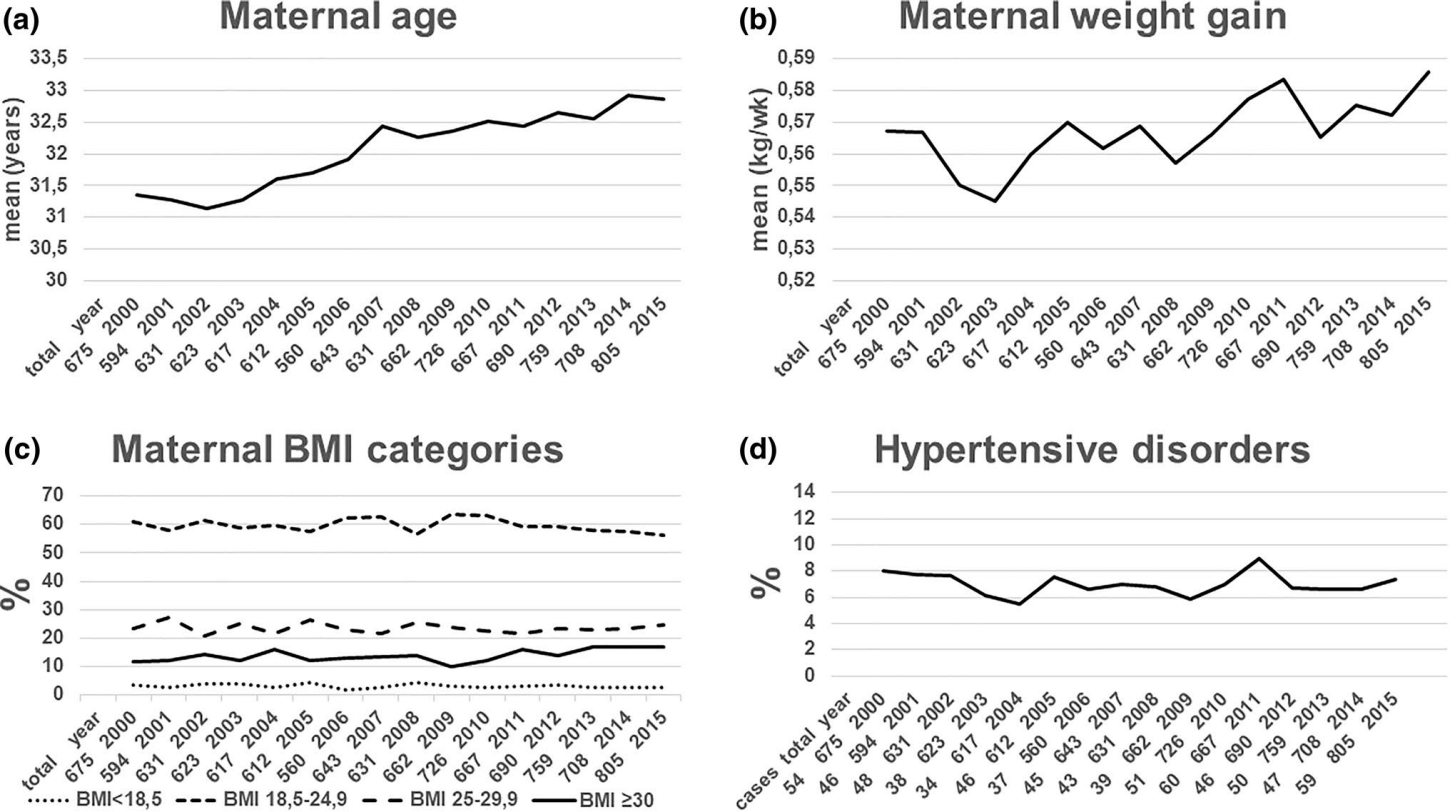
Course of maternal outcomes between 2000 and 2015, *n* = 10,603, total = total twin deliveries, cases = absolute incidence of specific outcome, + = positive correlation, − = negative correlation, statistical analysis by linear (**a**, **b**) or logistic (**c**, **d**) regression. **a** Mean maternal age (years): *p* < 0.0001+, **b** mean maternal weight gain/gestational week (kg/week): *p* = 0.001+, **c** maternal body mass index categories (%) at 1st examination (< 14 gestational weeks): underweight: *n* = 324, *p* = 0.14; normal weight: *n* = 6321, *p* = 0.11; overweight: *n* = 2489, *p* = 0.62; obese: *n* = 1469, *p* < 0.001+, **d** hypertensive disorders in pregnancy (%): *n* = 743, *p* = 0.77

As shown in Fig. 2a, the mean maternal age rose significantly between 2000 and 2015 from 31.4 to 32.9 years (*p* < 0.0001, Fig. 2a) and showed an even larger increase among primiparous women: from 30.8 years in 2000 to 32.9 years in 2015 (*p* < 0.00001). The other maternal parameters are shown in Fig. 2c, d. The mean maternal GWG increased from 567 to 586 g/week (*p* = 0.001, Fig. 2b).

The cutoff for a “low gestational weight gain” defined as below the 25th quartile was 419 g/week and for “excessive gestational weight gain” defined as above the 75th quartile was 692 g/week. Although a growing absolute and relative number of women had a BMI of 30 kg/m^2^ or higher (*p* < 0.001) at their first examination, there was no significant change in the rates of underweight women (*p* = 0.14) (Fig. 2c). The rates of HDP did also not change significantly between 2000 and 2015 (*p* = 0.77, Fig. 2d).

**Fig. 3:**
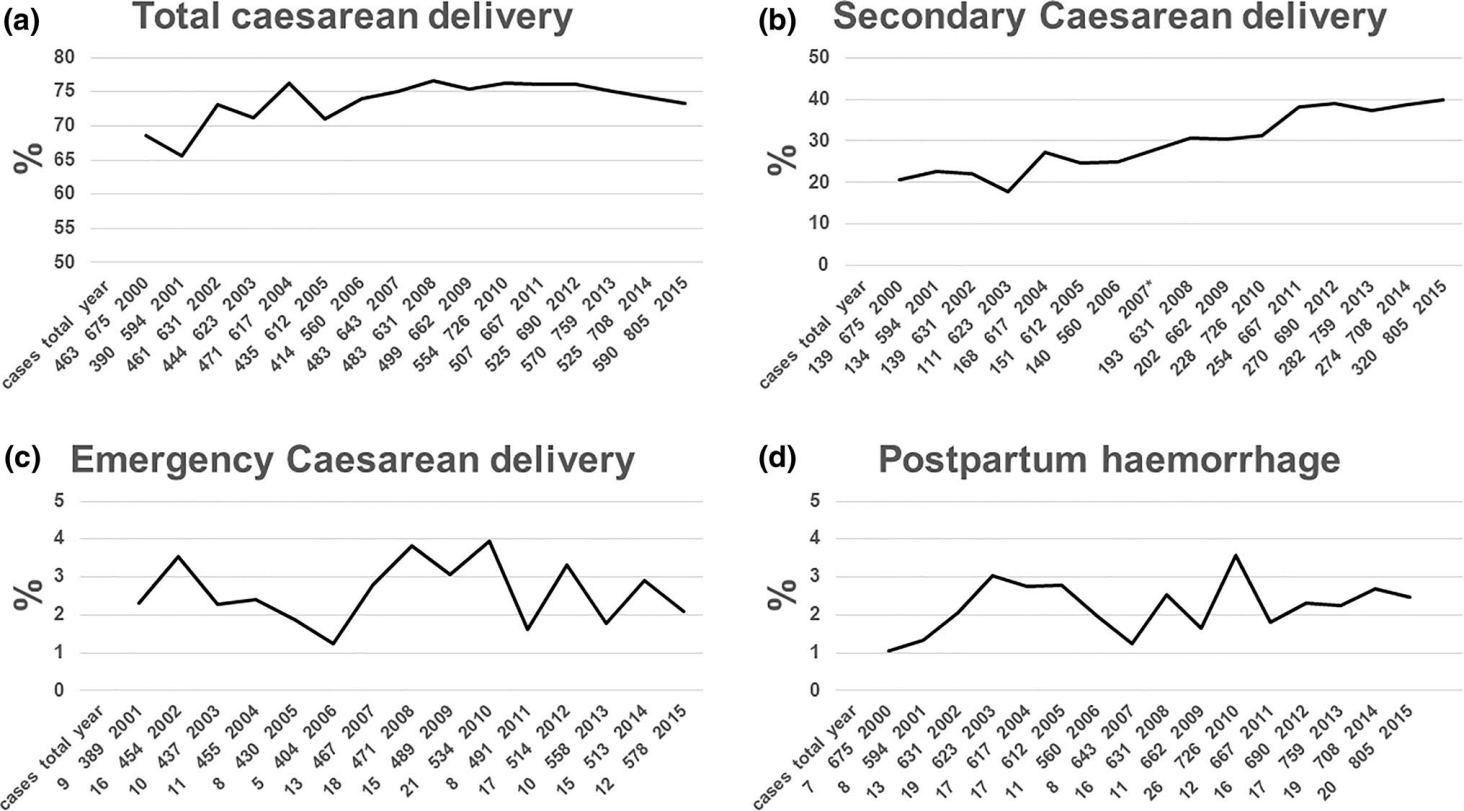
Course of caesarean deliveries and postpartum haemorrhage between 2000 and 2015, *n* = 10,603, total = total twin deliveries, cases = absolute incidence of specific outcome, + = positive correlation, − = negative correlation all statistical analyses by logistic regression. **a** Total caesarean delivery (%): *n* = 7814, *p* < 0.001+, **b** secondary caesarean delivery (%): *n* = 3 005, *p* < 0.001+, *data from 2007 was excluded due to missing values. **c** Emergency caesarean delivery (%): *n* = 188, *p* = 0.93 (no data available in 2000). **d** Postpartum haemorrhage > 1000 ml (%): *n* = 237, *p* = 0.12

Figure 3a–d shows the course of total, secondary and emergency caesareans and of PPH.

There was a significant increase of total and secondary caesarean deliveries from 68.6 and 20.6% in 2000 to 73.3% and 39.8%, respectively, in 2015 (*p *< 0.001, Fig. 3a, b). No significant change was observed in the rates of emergency caesarean delivery (*p* = 0.93, Fig. 3c) and in the rates of PPH (*p *= 0.12, Fig. 3d).

Apart from these figures we analyzed the rates of caesareans only in the second twin, e.g. combined delivery.

In 2000, this rate was 3/675 (0.4%), but increased up to 20/726 (2.9%) in 2010 in only 10 years (*p* < 0.01).

**Fig. 4:**
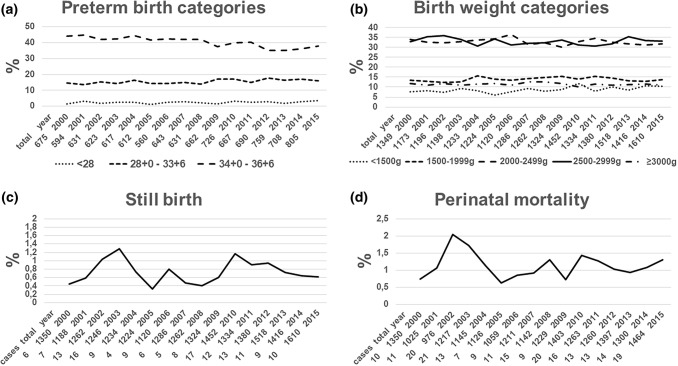
Course of neonatal outcomes (I) between 2000 and 2015, *n* = 21,206, total = total twin deliveries (**a**) or newborn twins (**b**–**d**), cases = absolute incidence of specific outcome, + = positive correlation, − = negative correlation, all statistical analyses by logistic regression. **a** Preterm deliveries (%) in categories: delivery < 28 weeks: *n* = 520, *p* < 0.01+; delivery between 28 + 0 and 33 + 6 weeks: *n* = 3301, *p* = 0.002+; delivery between 34 + 0 and 36 + 6 weeks: *n* = 8548, *p* < 0.001−. **b** Birth weight (%) in categories: < 1500 g: *n* = 1860, *p* < 0.001+; 1500–1999 g: *n* = 2941, *p* = 0.41; 2000–2499 g: *n* = 6903, *p* = 0.07; 2500–2999 g: *n* = 6958, *p* = 0.33; > 3000 g: *n* = 2413, *p* = 0.50. **c** Stillbirth (%): *n* = 155, *p* = 0.85. **d** Perinatal mortality (%): *n* = 221, *p* = 0.82

**Fig. 5:**
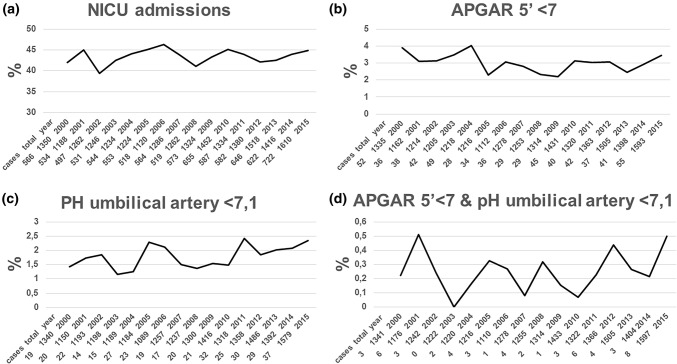
Course of neonatal outcomes (II) between 2000 and 2015, *n* = 21 206, total = total newborn twins, cases = absolute incidence of specific outcome, + = positive correlation, − = negative correlation, all statistical analyses by logistic regression. **a** NICU admissions (%): *n* = 9213, *p* = 0.28. **b** Newborns with an APGAR below 7 after 5 min (%): *n* = 633, *p* = 0.16. **c** Newborns with a pH in the umbilical artery below 7.1 (%): *n* = 370, *p* = 0.03+. **d** Newborns with an APGAR 5′ below 7 and a pH in the umbilical artery below 7.1 (%): *n* = 53, *p* = 0.39

The examined neonatal outcomes are illustrated in Figs. 4 and 5, including the categories of preterm birth and BW. The rates of early preterm birth (< 28 and 28 + 0 to 33 + 6 weeks) (*p* < 0.01, Fig. 4a) and newborns with a BW < 1500 g (*p* < 0.001, Fig. 4b) all increased. Rates of stillbirth (*p* = 0.85, Fig. 4c), perinatal mortality (*p* = 0.82, Fig. 4d), NICU admissions (0.28, Fig. 5a) or APGAR scores after 5 min below 7 (*p* = 0.16, Fig. 5b) did not significantly change between 2000 and 2015. Even worse, in spite of increasing caesarean rates, the rates of twins with a pH in the umbilical artery at birth below 7.1 did not decrease, but increased during the observation period (*p* = 0.03, Fig. 5c).

## Discussion

### Principal findings

Between 2000 and 2015, twinning rates in this German cohort were rising from 1.5 to 1.9% (29%) parallel to a rise of maternal age from 31.4 to 32.9 years (4.8%) and of a BMI from 24.4 to 25.4 kg/m^2^ (4.1%). Similarly, there was a 3.3% increase of mean GWG from 567 to 586 g/week between 2000 and 2015. The increasing rates of caesareans were associated with an increase of early preterm birth, twins with a BW < 1500 g and increased rates of acidosis (pH < 7.1 in the umbilical artery) and were not accompanied with a significant change in stillbirths, perinatal mortality or admission of the twins to a NICU.

### Meaning of the findings

An *increasing twinning rate* is also observed in other countries and continents [9, 16–19] up to date. Maternal age, weight, genetic factors and artificial reproductive technologies (ART) are the main determinants of twinning and higher-order multiple rates [20]. To compensate overenthusiastic use of ART, several countries established regulations to reduce an uncontrolled origin of twin and higher-order multiple pregnancies caused by ART [21].

But even within Europe, legislation and practice vary considerably [22] resulting in rates of deliveries after ART between 0.54% in Malta and 4.1% in Denmark [23] which may impact the country-specific twinning rates. The policy of single versus double or multiple embryo transfer also plays an important role. It was shown that electively choosing single blastocyst transfer can significantly reduce the risk of multiples among women < 40 years without compromising their pregnancy rates [24]. Between 2006 and 2017, the total fertility rate in Europe ranged between 1.54 and 1.61 children/woman [25] although a mean of 2.1 children/woman is considered to keep the population stable in developed countries [26]. Therefore, health-care politicians and health-care providers have to responsively balance and set their political priorities accordingly.

Similarly to the *rising maternal age* in this German cohort, the mean age of women at childbirth in 28 countries of the EU increased from 29.6 years in 2006 to 30.7 years in 2017 [27]. Older women are more likely to have twins, a high BMI and to be delivered by a caesarean [28, 29] which explains the inter-relationships of our results. Similar developments have been described:

With respect to the *delivery mode* the EURO-PERISTAT study already reported on large varieties in caesareans within Europe for different indications [30]. For multiple gestations, the rate of caesareans varied from north to south with 31.1% in Iceland to 98.6% in Malta. In central Europe, the rates were 43.9% in the Netherlands, 54.8% in France and 74.8% in Germany. Studies considering changing rates over time were published from Israel, whereby the authors reported on a total increase of caesarean rates in twins from 43.4% in 1995 to 66.0% in 2015 with increasing numbers only in non-laboring women [31]. In our cohort, primary caesareans did not increase. Similarly to our results, the increasing caesarean rates in Israel were not combined with lower rates of Apgar score < 7 at 5 min or of PPH during this period [31].

In the US, caesarean rates in twins increased from 53.4% in 1995 to 75.0% in 2008. It was worrisome that these were not indicated by higher rates of obstetric or medical complications [32]. The total increase of caesareans in twin pregnancies in Western countries and the significant increase of secondary caesareans in this German cohort are most probably caused by a lack of skills in vaginal twin delivery and the fear of malpractice cases: Accordingly, we found an increase of caesareans in only the second twin within our cohort from 3/675 (0.4%) to 20/726 (2.8%). A survey in the US stated that despite national guidelines that encourage vaginal twin birth, concerns remain about the availability of skilled obstetricians [33]. Therefore, we have published detailed teaching programmes on this issue [34, 35]. We also plan to perform a survey among residents based on the same evaluated questionnaire as by Easter et al. [33] and then to provide focused interventions of simulation-based training and provider support to increase obstetricians’ comfort with vaginal twin vaginal delivery (work in progress, supported by the Clara Angela Foundation and the German AG Twin Pregnancy).

Since *excessive gestational weight gain* (EGWG) has been identified as another risk factor for caesarean delivery rates [7, 36], the increase of EGWG may also explain the rising rates of caesareans in our cohort. Therefore, the here given quartiles could be of tremendous help for clinicians to avoid accelerated or decreasing weight gain in mothers of twins even within weekly controls. Although several studies have shown that EGWG and a high pre-pregnancy BMI lead to higher rates of HDP and preeclampsia [7, 8, 37, 38], this could not be demonstrated in our cohort which might have partly be caused by the indirect determination of HDP.

Studies on the effect of maternal weight on *preterm births* in singleton pregnancies are contradicting: in two studies overweight and obese women had increased risks of provider-initiated preterm delivery, but decreased risks of spontaneous preterm births [39, 40]. However, several studies consistently showed that in twin pregnancies a high maternal BMI increases the risk for all kinds of preterm deliveries [41, 42], and one study indicated that this was even the case for spontaneous preterm births [43]. The same association between maternal overweight or obesity and preterm births has been proven among twin pregnancies conceived by ART [44]. Thereby, a high maternal age does not seem to have a significant impact on the rates of preterm birth in twin pregnancies [45] and was even discussed to decrease the risk [46]. Whether the increasing rates of preterm deliveries in our cohort can be explained by a high BMI should be analyzed by multivariate regression models.

With respect to the *birthweight of twins* our results suggest that the rate of twins with a BW < 1500 g increased in parallel with increasing pre-pregnancy BMI and EGWG, which is in contrast to other results [8, 36, 47]. Pre-pregnancy maternal underweight and low maternal GWG are reported to be associated with higher rates of preterm birth, low BW or SGA and NICU admissions [37, 48–52]. In our cohort, the rate of underweight women did not change with time and thus can hardly explain the increasing rates of low BW and prematurity in twins. Therefore, it might still be suspected that iatrogenic early caesareans might have led to increasingly unfavourable outcomes.

*Long-term maternal and neonatal outcomes* are strongly influenced by high maternal BMI and EGWG in singleton pregnancies showing higher risks for postpartum weight retention and cardiovascular events for the mothers [2, 3, 53] as well as higher rates of childhood obesity and even lower life expectancy in the offspring [4, 54]. However, the effects have not yet been demonstrated specifically for mothers of twins and their offspring and should therefore be analyzed in future trials.

### Clinical implications

In general, women need to be informed about the risks of twin pregnancies and the consequences of a deviating BMI or GWG for both, mother and their offspring which differ from singleton pregnancies. Specific results cannot be concluded from only descriptive analyses, but require multivariate analyses (work in progress).

The IOM guidelines have proven to be an evidence-based framework to help in the surveillance of maternal weight gain for singletons [6]. Even though the recommendations for twin pregnancies were only provisional and related to the whole duration of pregnancy, some studies showed that women with an adequate as compared to women whose total weight gain was less than recommended had lower rates of SGA and preterm birth, whereas no significant improvement was shown when data were compared with women of EGWG [50, 55, 56]. Therefore, the pragmatic cutoffs we here provide for weekly weight gain in the form of quartiles may already enable obstetricians to identify unusually high or low GWG and to provide better counseling for women pregnant with twins. Presently, we perform multivariate analysis models to further evaluate the impact of both insufficient and excessive GWG on maternal and neonatal outcomes in twin pregnancies.

### Strengths and weaknesses

The strength of the study is that the cohort of twin pregnancies is large enough to demonstrate significant changes during a time period of 15 years which allows to compare regional and national trends in comparison to other German or European cohorts. In addition, the size of the total cohort was large enough to calculate quartiles of weight gain during twin pregnancy which were not yet provided to clinicians before.

Our study also has weaknesses: data from perinatal registers in Germany are not sufficiently controlled in terms of completeness of data and plausibility. Therefore, it took us 1 year to correctly select only plausible data. Nevertheless, data on GDM appeared retrospectively unreliable in terms of method and gestational age of diagnosis. This is why we did not further analyze GDM in our cohort. However, there are recent data that in contrast to singleton pregnancies, GDM in twin pregnancies is not associated with HDP or pour neonatal outcome but with accelerated fetal growth, which later might change the metabolic morbidity [57].

Another weakness is that the registry did not allow differentiating between mono- and dichorionic twins or between the origin of pregnancy. Thus we were not able to determine the individual impact of ART on the increase of twinning rates and the outcome of chorionicity and ART pregnancies as compared to spontaneous origin.

Not analyzing a causal relationship between maternal weight characteristics in pregnant mothers of twins and the outcome could be regarded as a weakness but this was intentiously postponed to work in progress.

## Conclusion

The global and national trends of the obesity epidemic were equally observed in this German cohort of twin pregnancies. In parallel, there was no improvement but even an aggravation of maternal and neonatal outcomes reflected by increasing rates of caesareans and of preterm birth, low BW and low pH values at birth between 2000 and 2015. This should increase awareness among policy makers and stimulate health-care providers to intensify counselling of pregnant mothers of twins. The here defined cutoff values for maternal weight gain in twin pregnancies are a chance to identify abnormal maternal weight gain. In addition, iatrogenic preterm caesareans and combined deliveries should be avoided and skills in vaginal delivery of twins improved.
